# Exposure to Toxic Metals and Health Risk Assessment through Ingestion of Canned Sardines Sold in Brazil

**DOI:** 10.3390/ijerph19137678

**Published:** 2022-06-23

**Authors:** Luana Carolina Santos Leite, Nayara Vieira de Lima, Elaine Silva de Pádua Melo, Carla Maiara Lopes Cardozo, Valter Aragão do Nascimento

**Affiliations:** Group of Spectroscopy and Bioinformatics Applied to Biodiversity and Health (GEBABS), Graduate Program in Health and Development in the Central-West Region of Brazil, School of Medicine, Federal University of Mato Grosso do Sul, Campo Grande 79079-900, Brazil; nayaralima.01@hotmail.com (N.V.d.L.); elainespmelo@hotmail.com (E.S.d.P.M.); nutricarlalopes@gmail.com (C.M.L.C.)

**Keywords:** heavy metals, metalloids, pollutant, processed fish

## Abstract

The presence of heavy metals in the environment is increasing, which can be a danger to public health. Fish exposed to contaminated environments tend to have higher concentrations of some metals in their tissues. Monitoring these elements remains urgent as it is a matter of global concern. Canned sardines from the Brazilian market were analyzed for elements (Al, As, Ba, Cd, Co, Cr, Cu, Fe, Ni, Pb, Se, and Zn) of metals and metalloids, including some toxic, using inductively coupled plasma optical spectrometry (ICP OES) in two types of sardines (preserved in oil and tomato sauce) from five different brands. The results were compared to limit levels for consumption set by FAO/WHO. Moreover, we assessed the associated risk regarding the elemental intake of these elements through the samples, using the hazard quotient (*HQ*), hazard index (*HI*), and carcinogenic risk (*CR*). All samples had unfavorable *HQ* and *HI*, primarily due to arsenic content. In the same manner, *CR* for arsenic was above the proposed limit of 10^−4^, and cadmium and chromium, which were within the acceptable limit (10^−6^ to 10^−4^), require attention. These results show that chronic consumption of canned sardines sold in Brazil is unsafe, and quality surveillance is needed to ensure there is no risk to the population that ingests these products.

## 1. Introduction

Heavy metal contamination in foods and aquatic ecosystems is considered a severe problem at the global level due to the effects on the environment and human health [1]. The non-essential elements, such as aluminum (Al), arsenic (As), lead (Pb), mercury (Hg), and cadmium (Cd), are harmful to organisms, even in small concentrations, due to their tenacity, nondegradability, biological toxicity, and capacity to enter the food chain [2]. Conversely, metals such as copper (Cu), chromium (Cr), and zinc (Zn) are considered essential because they perform physiological functions in human health when their intake is adequate; however, at higher concentrations, their risk cannot be underestimated [3].

Therefore, although metals occur naturally in nature, the increasing pollution from human activity leads to rising elemental concentrations in the marine environment and coastal areas [4]. Contamination by humans occurs via industrial (fossil fuel combustion, metal processing), agricultural, and domestic residues as garbage and detergents [5]. These metals and non-metals are dissolved in water columns and sediments through fine surface particles that can accumulate in living marine organisms, such as phytoplankton [4]. In this context, small pelagic fishes have a vital importance for food webs, feeding on plankton and serving as a food source for various marine species of fish, seabirds, dolphins, and marine mammals [6]. In addition, marine organisms may absorb from surrounding areas [3,7], and present a direct connection between heavy metal pollution and the health of coastal residents [8].

The progression of metal and non-metal contamination is of interest because of the direct adverse effects on exposed organisms, but also indirectly due to the consumption of food contaminated with toxic concentrations of these elements [9]. The damage to aquatic ecosystems and consumer health can be significant since these metals in water often occur in chemical forms that are more easily absorbed, can bioaccumulate, and can be biomagnified through the food chain until they reach humans [3,9,10].

Nonetheless, eating habits that include fish are associated with health, longevity, and lower incidence of cardiovascular disease, neurodegenerative diseases, and cancer [11]. Other benefits include reduced insulin resistance and control of type 2 diabetes mellitus, and reduced obesity, depression, metabolic syndrome, and inflammatory diseases [12,13,14,15]. Hence, the American Heart Association (AHA) recommends a weekly intake of 2 to 3 servings of fish and seafood to ensure the necessary doses of polyunsaturated fatty acids (PUFAs), such as omega-3, associated with cardioprotection [16].

The World Health Organization (WHO) recommends a minimum consumption of fish of 12 kg/habitant/year [17,18]. In 2018, the State of the World Fisheries and Aquaculture (SOFIA) estimated the world average per capita fish consumption to be 20.5 kg/habitant/year, whereas Brazilians ate an estimated 9.5 kg/year habitant in 2020 [17].

Canned fish is an alternative used worldwide for the preservation and increased shelf life of fish products; however, such products present a higher risk of contamination of trace elements coming from the species of fish used in the canning and the material used in the packaging, which is a kind of steel can coated with electrolytic chromium/chromium oxide [19].

Studies from several countries have determined the content of inorganic contaminants in canned fish, suggesting threshold concentrations and detecting excessive inorganic contamination, serving as parameters to avoid carcinogenic and neurotoxic effects on the human organism [19,20].

Therefore, this study aimed to quantify the concentration of essential and toxic elements in canned sardine samples commercialized in Brazil of high consumption and commercial interest; to evaluate the risk to human health due to the ingestion of aluminum (Al), arsenic (As), barium (Ba), cadmium (Cd), cobalt (Co), chromium (Cr), copper (Cu), iron (Fe), nickel (Ni), lead (Pb), selenium (Se), and zinc (Zn) in canned sardine samples: (i) in oil (SO), and (ii) in tomato sauce (ST); and to assess the accumulation of heavy metals through sardine consumption in different age categories, i.e., toddlers, children, adolescents, and adults. The results show that all samples are unfit for consumption due to elevated levels of the hazard quotient (*HQ*), hazard index (*HI*), and carcinogenic risk (*CR*).

## 2. Materials and Methods

### 2.1. Sample Collection and Preparation

A total of 120 samples of canned sardines were purchased from five Brazilian companies from supermarkets in Campo Grande, Mato Grosso do Sul, Brazil. There are five brands of major national market companies that sell these two types of canned sardines in Brazil, and the sample groups were divided into 60 samples of sardines in oil (SO) and 60 samples of sardines in tomato sauce (ST). Samples were identified with the group to which they belong (SO or ST), and each of the five brand codes (G, C, O, P, and Pa).

The collected packages, still closed, were sanitized with neutral detergent and rinsed with demineralized water (ELGA, Veolia Water Solutions & Technologies, Houston, TX, USA). After opening, the liquid content (oil or tomato sauce) was drained with a plastic sieve. The meat was placed into identified and sterilized glass beakers. The meat content was ground in a food blender with stainless steel cutters. About 400 mg of the sardine tissue was weighed and then digested in 1 mL of nitric acid (65%), 1 mL of hydrogen peroxide (35%), and 3 mL of ultrapure deionized water. All standard solutions and chemicals were analytical grade from Merck (Darmstadt, Germany). The digestion procedure occurred in a microwave system (Speedwave four^®^, Berghof, Germany) in triplicate according to operating conditions shown in Table 1. After the digestion process, each tube had an addition of 5 mL of ultrapure water to its total volume for acidity adequacy.

Elemental determination in samples was performed using an inductively coupled plasma optical emission spectrometer (ICP-OES) with an axial view (iCAP 6300 Series, Thermo Scientific, Waltham, MA, USA). The instrumental and operating parameters for ICP-OES are shown in Table 2.

The calibration curves for all the analytes were constructed on nine different concentrations over the range of 0.005–2 mg/L. A multielement solution containing 100 mg/L Al, As, Ba, Cd, Co, Cr, Cu, Fe, Ni, Pb, Se, and Zn (SpecSol-Quinlab, São Paulo, Brazil) of each element was used to construct calibration curves.

The calculation of the limits of detection (LOD) and limits of quantification (LOQ) was according to the analytical standards established by the Union of Pure and Applied Chemistry (IUPAC) [21]. Table 3 shows the calibration curve parameters, LOD and LOQ values, and the obtained correlation coefficient (R^2^).

An addition/recovery test was used for validating and checking the accuracy of the analytical techniques used by spiking (0.5 mg/L of each analyte). The results show that the method has good accuracy and a 95–117% recovery interval, showing no systematic errors or loss of elements during the digestion process [22].

### 2.2. Human Health Risk Assessment

Risk analysis for non-carcinogenic chronic damage was performed for macro- and microelements, essential and toxic, using the method described by Lima et al. [23].

To calculate the risk to human health from the ingestion of heavy metals, the chronic daily intake (*CDI*) dose for each inorganic contaminant in canned sardines was initially evaluated, considering a period of exposure and the amount of fish ingested during this period. *CDI* values were expressed in (mg/kg/day) and calculated using Equation (1):

(1) $$CDI=\frac{C_{sardine}\times IR_{sardine}\times EF\times ED}{BW\times AT}$$

where: *C_sardine_* is the concentration of each element detected in canned sardine samples (mg/kg) [24]; *IR_sardine_* is the daily intake rate, standardized in this study as the portion of sardines per can standard in Brazil (84 g/dia); *EF* é is the exposure frequency (3 times per week, totaling 156 days/year) as recommended by the FDA and EPA [25]; *ED* is the duration of this exposure considering the following groups: a child with exposure aged two years, aged five years, teenager aged 14, and adult aged 30 years; BW is body weight (kg), 12 kg for 2-year-olds; 23.1 kg for 5-year-olds; 52 kg for a 14-year-old, and 70 kg for a 30-year-old adult, as established by the European Food Safety Authority (EFSA) [26]; AT is the average exposure time converted into days (*AT* = *ED* × 365 days/year). The average daily consumption was set at 84 g/day as it is the amount referring to a can of sardines, although this portion is well below the weekly recommendation of 2 to 3 portions of 226 to 340 g [25].

The hazard quotient (*HQ*) level was calculated starting from the *CDI* values for chronic non-carcinogenic damage in humans from the ingestion of fish contaminated with heavy metals, where the *HQ* is a ratio between the *CDI* and the chronic oral reference dose (*RfD*); Equation (2):

(2) $$HQ=\frac{CDI}{RfD}$$

The *RfD* is the maximum tolerable daily intake of a metal(loid) without harm to health, and was established by the United States Environmental Protection Agency (USEPA), in the “Regional Screening Levels (RSLs)—Summary Table”, having the following values for the relevant elements: Al = 1.0 mg/kg/day; As = 0.0003 mg/kg/day; Ba = 0.2 mg/kg/day; Cd = 0.0001 mg/kg/day; Cr = 0.003 mg/kg/day; Cu = 0.04 mg/kg/day; Fe = 0.7 mg/kg/day; Ni = 0.011 mg/kg/day; Pb = 0.0035 mg/kg/day; Se = 0.005 mg/kg/day; Zn = 0.3 mg/kg/day [27].

When *HQ* values are above 1, it indicates potential damage associated with the analyzed element, whereas *HQ* < 1 does not indicate risk [28,29].

Another parameter related to the *HQ* is the hazard index (*HI*), which is the sum of the risk quotients for an individual simultaneously exposed to two or more metals, obtained through Equation (3):

(3) $$HI=HQ_{Al}+HQ_{As}+HQ_{Ba}+HQ_{Cd}+HQ_{Cr}+HQ_{Cu}+HQ_{Fe}+HQ_{Ni}+HQ_{Pb}+HQ_{Se}+HQ_{Zn}$$

If *HI* < 1, the intake dose regarding these elemental contents is safe; however, consumption may pose a health risk in the case of *HI* > 1 [29].

Since the US EPA lists inorganic arsenic as a human carcinogen [30,31], it is possible to estimate the carcinogenic risk (*CR*), which is the probability of an individual developing cancer during their lifetime due to exposure to a chemical known to be carcinogenic. The *CR* for arsenic was calculated through daily exposure over the years of life for toddlers, children, adolescents, and adults, using the following Equation (4):

(4) Carcinogenic Risk=CDI×SF

where the *CDI* is the daily dose of chronic ingestion of carcinogenic elements (mg/kg/day), and the slope factor (*SF*) is the slope factor of this carcinogenic chemical element (mg/kg/day), which results from a low extrapolation dose, by which the risk of accumulated exposure is determined [32].

Arsenic and the other three heavy metals were considered carcinogenic and their available SF values are the following: As = 1.5 mg/kg/day; Cd = 6.1 mg/kg/day; Cr = 0.5 mg/kg/day; Pb = 0.0085 mg/kg/day [32,33,34]. According to the US EPA [35], the acceptable cancer risk value ranges from 10^−6^ to 10^−4^, considering the amount from different exposure routes, so the sum of values with concentrations greater than 10^−4^ is considered unacceptable.

### 2.3. Comparative Criteria

Initially, the results of the concentration of macro- and microelements of the SO and ST sardine samples were compared to the concentrations of previous national and international studies in the 84 g portion of canned sardines. They were compared with the references of maximum tolerable intake level (UL) for adults [36], provisional tolerable weekly intake (PTWI), tolerable daily intake (TDI), provisional maximum tolerable daily intake (PMTDI), and maximum tolerated limit (MTL) [37,38].

### 2.4. Statistical Analysis

The data were analyzed by two-way ANOVA using the GraphPad Prism 8 software version 8.0 (GraphPad Software, San Diego, CA, USA). One-way ANOVA was used to detect the difference between sample elemental quantification, and two-way ANOVA was used to detect the variation source. The sources of variation considered were sample brands and type of preservation (oil or tomato sauce). The significance of the differences between the means for the individual trace element was considered at *p* < 0.05.

## 3. Results

### 3.1. Elemental Content in Canned Sardine Samples

An overview of the concentrations of the different elements analyzed is displayed in Table 4. In this study, concentrations of metals (Al, As, Ba, Cd, Cr, Cu, Fe, Ni, Pb, and Zn), a non-metal (Se), and a metalloid (As) were detected in samples of sardines canned in oil and tomato sauce. Otherwise, cobalt levels in all samples were below the limit of detection.

Aluminum content in canned ranged from 0.001 to 0.008 mg/84 g. Two-way ANOVA showed that brand, conservative type, and interaction played a role in aluminum content. The brand was responsible for the major part of the variation (53.92%, *p* < 0.0001), followed by the interaction (34.13%, *p* < 0.0001), and conservative type (7.79%, *p* = 0.0015).

The results obtained from arsenic in all brands of sardines in oil (SO) ranged between 0.256 and 0.377 mg in an 84 g portion, and the samples of sardines preserved in tomato sauce (ST) varied between 0.207 and 0.323 mg/84 g. Arsenic levels did not depend on the conservative type alone (*p* > 0.05), whereas the brand was the more significant variant regarding this metal content (44.59%, *p* = 0.0127).

The contents of barium found in the SO samples were between 0.020 and 0.048 mg/84 g, whereas the ST samples presented higher barium concentrations than the oil version, of between 0.050 and 0.1377 mg/84 g. The major responsible component for element variation was the interaction between brand and conservative type (51.24%, *p* < 0.0001).

Cadmium detection ranged from 0.00002 to 0.002 mg/84 g in samples. According to two-way ANOVA results, brand, conservative type, and interaction are responsible for variation in the metal content; where the interaction played the most important role (46.08%, *p* < 0.0001), followed by brand (42.92%, *p* < 0.0001), and conservative type (10.67%, *p* < 0.0001).

Nickel was quantified only in the samples SO-C (0.009 mg/84 g) and ST-O (0.0002 mg/84 g). Neither brand, conservative type, or interaction were crucial to the nickel content variation.

Chromium concentration in samples ranged from 0.0003 to 0.0024 mg/84 g, whereas the ST-P sample did not have a detectable amount of this element. Chromium content did not differ due to brand (*p* = 0.9624), conservative type (*p* = 0.2145), or interaction (*p* = 0.9434).

Results for copper contents for all samples were between 0.0544 and 0.2205 mg/84 g. Although the interaction between brand and conservative type was the principal component in the metal amount variance (41.15%, *p* = 0.0248), the brand was responsible for 35.05% (*p* = 0.0390), and the conservative type alone was not enough to make a difference considering copper content (0.8541%, *p* = 0.5554).

The minimum iron level in samples was 1.463 mg/84 g, and the maximum level was 3.507 mg/84 g (Table 4). This variation did not depend specifically on brand (*p* = 0.4258), conservative type (*p* = 0.3568), or interaction (*p* = 0.0848).

Lead detectable content in oil and tomato sauce samples varied between 0.00007 and 0.0009 mg/84 g. The primary factor for lead content was the interaction (43.51%, *p* < 0.0001), followed by brand (43.09%, *p* < 0.0001), and conservative type (10.92%, *p* < 0.0001).

Selenium contents shown in Table 4 for the SO samples are between 0.172 and 0.191 mg/84 g, whereas the ST group presented minimum and maximum values of 0.157 and 0.203 mg/84 g, respectively. No factors played a significant role in selenium variation (brand-p = 0.8464; conservative type-p = 0.4254; or interaction, *p* = 0.5657).

The zinc values in the bottom half of Table 4 range from 0.0171 to 0.0391 mg/84 g, considering all samples. The principal component for zinc content variation between samples was the interaction (38.13%, *p* = 0.0449) between brand and conservative type; neither brand or conservative type impacted this elemental content in a significant manner (*p* = 0.0539 and *p* = 0.9432, respectively).

### 3.2. Elemental Distribution

**Figure 1:**
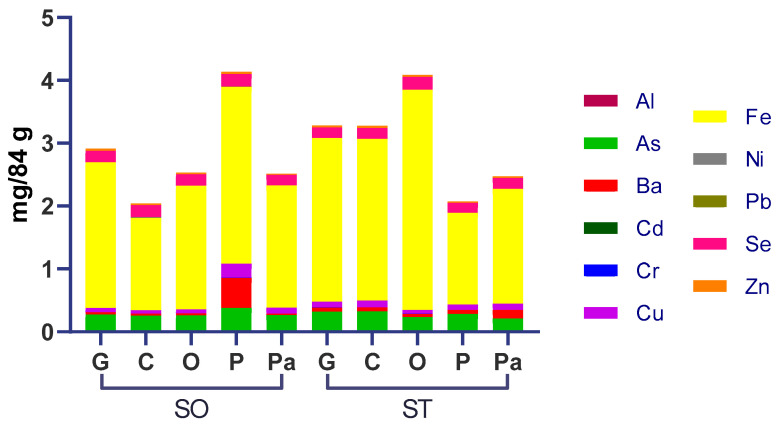
Elemental content distribution in canned sardines quantified by ICP OES.

As can be seen below, Figure 1 provides an overview of the elemental distribution among samples.

Iron was the most predominant element detected across all samples. Other prevalent metal(loids) were selenium and arsenic. The sample SO-O had a divergent elevated barium amount.

### 3.3. Human Health Risk Assessment

The risk assessment results for the different population groups were found to vary according to age.

**Figure 2:**
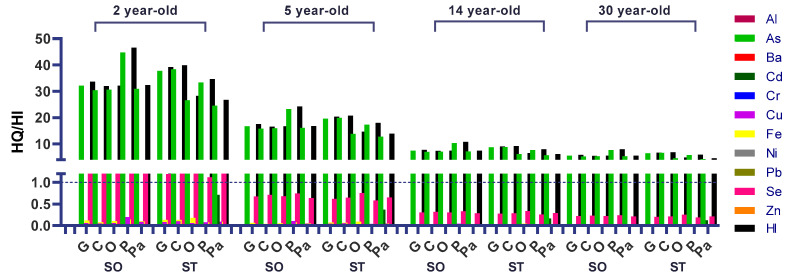
Risk quotient (*HQ*) and hazard index (*HI*) due to the consumption of canned sardines sold in Brazil for individuals aged 2, 5, 14, and 30 years.

Figure 2 describes the risk quotient (*HQ*) and hazard index (*HI*) values for Al, As, Ba, Cd, Cr, Cu, Fe, Ni, Pb, Se, and Zn, considering the consumption of canned sardines for toddlers, children, adolescents, and adults.

From Figure 2 below, the As group reported significantly more risk ratios than the other element groups. Moreover, all risk ratios for As in sardines canned in oil and tomato sauce of the five brands analyzed were above 1 for toddlers, children, adolescents, and adults. This result is contraindicated and raises concerns.

According to the non-carcinogenic risk index (*HI*) calculated by adding up the risk quotients for simultaneous exposure to metals, all SO and ST samples showed an *HI* value greater than 1 for all population groups studied.

The data presented in Table 5 demonstrate the carcinogenic risk (*CR*) results for As, Cd, Cr, and Pb exposure by eating canned sardines from five Brazilian brands for toddlers, children, adolescents, and adults.

## 4. Discussion

### 4.1. Canned Sardines Elemental Content and Intake Limits

In Brazil, De Mello Lazarini et al. (2019) [19] found greater amounts of aluminum in canned sardines than we reported in Table 4, with concentrations between <0.04 and 1.112 mg/84 g. Migration aluminum levels assessed in simulant environments had higher amounts when in an acid solution than in an oil solution [39], which can partially explain the role of tomato sauce in aluminum distribution among samples. This data can best be classified under the maximum allowable limit of aluminum intake from food and water set by WHO at 2 mg/kg/week, equivalent to 17.71 mg/day for a 62 kg adult, 19.99 mg/day for 70 kg adults, and 7.43 mg/day for 26 kg children [40]. Although aluminum is widely distributed in the environment, it has no known biological function in humans, and high exposure to this metal has been linked to adverse health effects, particularly anemia, bone disease, and dialysis encephalopathy [41,42,43], whereas chronic exposure has been related to an increased risk of neurodegenerative disorders, including dementia, Parkinson’s and Alzheimer’s disease [44]. Nonetheless, all samples are within the recommended concentration.

Arsenic concentrations were higher in comparison to sardines preserved in oil marketed in the USA, with values of 0 to 0.00009 mg/84 g [45]. Again, our findings are above those from sardines sold in Poland, with an average of 0.162 mg/84 g of As [46], but comparable to other samples in Brazil, with arsenic levels ranging from 0.064 to 0.541 mg/84 g in oil, and from 0.055 to 0.351 mg/84 g in tomato sauce [19]. Consistent data shows that long-term exposure to arsenic from food can cause cancer [47] and neurodegenerative diseases, liver and cardiovascular disorders, and cytotoxic and genotoxic effects [48,49]. For this reason, there is no UL for arsenic because there is no safe value for consumption; thus, FAO/WHO committee withdrew the weekly consumption limit of 0.021 mg/kg/day, because it was considered unsafe at this previous level [50]. In addition, since there is no certainty regarding the dose–response relationship, the EFSA did not consider it safe to determine a limit value for daily or weekly intake [51]. Even with these concerns, some countries, such as Brazil, have established limits for consumption, where the maximum tolerated limit (MTL) for arsenic contamination in fish is 1 mg/kg [37]. Thus, all samples presented in Table 4 exceeded the threshold of this contaminant. This information is necessary because it indicates that, globally, certain regions may allow their population to be at risk; even considering the MTL for Brazil, this limit was exceeded.

The acidity of the tomato sauce may explain the fact that ST samples had a higher barium content than the oil-conserved samples. Demont et al. (2012) [52] reported a higher metal migration from a ceramic container surface with decreased pH for most metals, including barium. Barium accumulation can occur by ingesting contaminated food and water, and there is no evidence that barium is carcinogenic or mutagenic. However, animal studies report the occurrence of nephropathic, neurological, cardiovascular, and metabolic diseases. In humans, it appears to have the potential to cause hypertension [53,54]. Although there is no established UL for barium, there are limit values of tolerable daily intake determined by FAO/WHO at 0.02 mg/kg/day for drinking water. This amount is equivalent to 0.52 mg/day for a child of 26 kg, 1.2 mg/day for an adult of 62 kg, and 1.4 mg/day for adults weighing 70 kg [54]; therefore, for toddlers and children, barium concentration in samples is considerably high. Although barium is a component of food cans, most of our samples did not show worrying levels of barium as a metallic contaminant. Agencies such as EFSA set a limit of 1 mg/kg for the migration of compounds from packaging to food [55], and thus only the ST-Pa sample exceeded this limit.

Brazilian sardines showed cadmium results between 0.0159 and 0.0319 mg/84 g [56], which were significantly above our findings. In another study in Brazil, cadmium content did not differ between conservative types (oil or tomato sauce) or between brands [19]. Nonetheless, the cadmium in these samples may be attributed to the levels of this element in the tomatoes, with possible contamination through the use of inorganic fertilizers [57,58]. Exposure to cadmium can lead to adverse effects, namely, renal and hepatic dysfunction, pulmonary edema, testicular damage, osteomalacia, and impairment of the adrenal and hemopoietic systems [59]. In Brazil, the Normative Instruction that regulates the maximum tolerated limit (MTL) of inorganic contaminants in foods determines cadmium concentrations of 0.008 and 0.021 mg/84 g for raw sardines and canned sardines, respectively [37]. According to international agencies, there is no limit set for cadmium intake [36].

The nickel quantification is in line with Kowaslka et al. (2020) [60] in canned fish, with a mean concentration of 0.088 mg/kg. Nickel occurs naturally in foodstuff because it is essential to plants. Oral intake is the most significant route for systemic absorption and may lead to toxicity in the general population; however, this route has no associated carcinogenic effects [61]. Regardless, nickel toxicity can occur, eliciting effects in several tissues and functions, and oral studies display effects on the liver, kidney, bone, gut microbiota, and the nervous and reproductive systems [62,63]. The nickel UL for adults over 19 years old determines the amount of 1 mg/day for soluble nickel salts only [64]. The tolerable daily intake (TDI) set for nickel intake is 13 µg/kg/day [63], so the samples all complied with this limit, and the chronic intake of canned sardines should not pose a risk regarding this element content.

The Brazilian study by De Mello Lazarini (2019) [19] compared chromium levels in different samples of sardines preserved in oil and tomato sauce. The results were highly similar, ranging from <0.0005 to 0.011 mg/84 g for oil and <0.0005 and 0.006 mg/84 g for tomato sauce, slightly higher than the values found in the present work. However, the levels observed in this investigation are far below those observed in Brazilian sardines, with the minimum and maximum contents of 0.038 and 0.099 mg/84 g, respectively [56]. The adequate intake for chromium is 15 µg/day for an 8-year-old child, 21 and 35 µg/day for female and male 18-year-olds, respectively, and 24 and 35 µg/day for female and male 30-year-olds, respectively [65]. There is no suggested value of PTWI/PMTDI determined by the JECFA nor a set UL [36,66]. It is essential to point out that there is no indication that trivalent chromium associated with food intake or supplements has caused adverse effects on a consistent matter [67]. Conversely, hexavalent chromium is linked to the development of certain types of cancer [68].

Safta et al. (2020) [39] found that both tomato sauce and oil conservatives rendered copper concentrations below the limits set for this element in canned sardines, agreeing with this experiment. Adequate doses of copper fulfill functions related to cardiovascular health, blood glucose regulation, and lipid metabolism [69]; it also has a regulatory effect on oxidative stress [70]. However, it causes mitochondrial dysfunction, liver damage, and Alzheimer’s disease in elevated amounts. Specific cases are more likely to suffer from excess or lack of copper; the case of Menkes syndrome can result in copper deficiency, and Wilson’s disease can occur due to toxicity [71,72]. Although there is no established UL for copper, FAO/WHO sets a PMTDI of 0.5 mg/kg/day for copper from all sources, equivalent to 35 mg/day for adults weighing 70 kg and 13 mg/day for children of 26 kg [73]. It is unlikely that this value could be achieved by consuming canned sardines for all samples.

Iron results contradict those found by Safta et al. (2020) [39], which determined elevated iron concentrations in tomato sauce0conserved sardines, which they attributed to the iron leaching from cans, provoked by the acidity and salt content in uncoated or flawed coated areas; in comparison, our study found consistent amounts in both tomato sauce and oil groups. An analysis by De Mello Lazarini et al. (2019) [19] quantified iron in samples preserved in oil and tomato sauce, with a detection range of 1.117 to 3.477 mg/84 g in oil-preserved samples and 1.26 to 3.528 mg/84 g in tomato sauce-preserved samples, in agreement with our findings. The daily intake of adequate iron levels guarantees acceptable serum concentrations of ferritin, associated with preventing anemia, which is still the most prevalent nutritional deficiency affecting children and adolescents worldwide [74,75]. The provisional maximum tolerable daily intake (PMTDI) established by FAO/WHO for iron is 0.8 mg/kg/day, equivalent to 56 mg/day for 70 kg adults and 20.8 mg/day for 26 kg children [76]. Moreover, the UL sets the iron limit of 45 mg/day for males, females, and pregnant women, which none of the samples exceeded; however, the iron concentration of the ST-O sample slightly exceeded the limit of 40 mg/day for children [36]. Hence, it is unlikely to cause harm to human health.

Our lead detection in samples is in agreement with De Mello Lazarini et al. (2019) [19], where lead concentrations in canned sardines sold in Brazil, preserved in oil and tomato sauce, varied between <0.0008 and 0.007 mg/84 g. Brazil adopted the European Food Safety Authority (EFSA) suggested limits in the maximum tolerated limit (MTL) of lead as a contaminant in fish of 0.025 mg/84 g [37,38]. Therefore, none of the samples exceeded the limits for contaminants in marine foods.

We did not find any factor contributing specifically to selenium variation, and De Mello Lazarini et al. (2019) [19] also found no difference among conservative types (tomato sauce or oil) in selenium detection; however, they did encounter a difference regarding the brand. Previous studies evaluating selenium observed inconsistent results for values of this element in canned sardine; the average content in Polish samples was 0.021 mg/84 g [46]; meanwhile, Turkish samples showed an average content of 0.233 mg/84 g of selenium [77], above that found in both SO and ST samples groups. Although rare, selenium toxicity can occur, leading to dermatitis, alopecia, high mortality, and cancer [78]. In addition to an excess, a deficiency is also harmful. Nonetheless, adequate daily doses help treat hypothyroidism and preeclampsia cases and may help prevent post-partum depression [79], diabetes mellitus, and neuropathies [78,80]. Concerning intake levels, all samples had selenium concentrations lower than the UL of 0.4 mg/day for adults and pregnant women, whereas for children, only the sample ST-P was within the limit of 0.15 mg/day [36]. Furthermore, studies set a daily selenium intake of 0.06 mg/day for pregnant women, which all samples provided [81].

Safta et al. (2020) [39] described that most of the zinc in canned sardine samples was due to migration from corrosion zones, particularly from bad tinning, and uncoated zones and joints in the can. Samples of sardines sold in Brazil in 2001 showed a range between 1.357 and 3.031 mg/84 g [56], much higher values than the analyses of Brazilian sardines in this work. Meeting the daily zinc needs as an essential element is pivotal in maintaining the organism’s homeostasis, growth, and development [82]; it also has also been proved to be necessary for the innate immune system, control of oxidative stress, and maintenance of macrophages [83]. The UL set for Zn for adults and pregnant women over 30 years old is 40 mg/day, whereas the limit for children is 5 mg/day [36]. Another critical parameter established by FAO/WHO is the PMTDI at 1 mg/kg/day [73]. All the brands showed values of Zn below the permissible limits set by UL and FAO/WHO. Therefore, the consumption of canned sardines seems safe for adults and children concerning zinc amounts in these samples.

### 4.2. Health Hazards Considering Elemental Intake through Canned Sardines Sold in Brazil

Selenium had *HQ* and *HI* above 1 for toddlers (2-year-olds) in all samples. Excessive nutrient intake at this age may be more harmful due to an incomplete development and the higher ratio between weight and intake, which could lead to anemia, nephrotoxicity, toxicity to the reproductive system, development impairment, lower intelligence quotient (IQ), and neurotoxicity [84]. The *HQ* for arsenic was above one for all samples and ages studied (Figure 2), and arsenic was the most significant contribution to the high levels of *HI*, posing a risk by itself. In addition, one worrisome piece of information is that arsenic was one of the most predominant quantified metals (Figure 1), showing high levels of arsenic contamination. Moreover, the carcinogenic risk was well above the threshold of 10^−4^ for all samples of all ages studied, reinforcing the hazard posed by this element alone in canned sardines sold in Brazil. Another fundamental fact to state is that only around 10% of arsenic in fish is inorganic, which is considered the highest toxic form [85]; however, even considering this amount, all *HQ* values would be above 1, and *CR* levels would be over 10^−4^, showing elevated levels of arsenic contamination in these samples.

Further, on carcinogenic risks, all other elements presented *CR*s within the acceptable limit of 10^−4^ but, besides arsenic, the second-highest risk was found for cadmium in ST-Pa samples for toddlers, children, and adolescents; whereas the lowest was for lead in ST-P and SO-Pa samples, both for adolescents and adults.

The *HQ* for cadmium was below 1 for all samples, suggesting a safe intake of this element for the canned sardines at the proposed amount of 84 g, three times/week. However, cadmium’s previous PTWI of 7 µg/kg/week was withdrawn by the Joint FAO/WHO commission for contaminants once they considered that no levels for cadmium ingestion are safe [86]; in this sense, any amount should be avoided.

It should be reinforced that any sample that has an associated cancer risk (current or future) greater than 10^−6^, as noted for cadmium and chromium, should remain on the list of chemicals of potential concern. Similarly, there are significant concerns about the sum of multiple contaminants [87].

In the same way, the *HQ* for lead is below one for all ages and samples, showing a low risk to lead chronic exposure. However, the determinations of JEFCA for the lead PTWI was withdrawn since even low doses of lead are no longer considered safe [76]. In this way, even small amounts of this metal may be prejudicial to the health status, and repeated exposures to the contaminant should be avoided.

For the elements aluminum, barium, chromium, copper, iron, nickel, selenium, and zinc, all *HQ* levels were below one in all samples taking into account the considered ages. These data reinforce the non-toxicity characteristic of the samples concerning the intake of those elements, which should not pose a health hazard regarding the proposed amounts and frequencies.

However, in addition to the health hazards, it is important to consider that contamination with toxic elements in fish tissue reflects increased metals in the water, whether by natural or anthropogenic pollution. The unprecedented increase in pollutants in the tissues of marine organisms worldwide [4,9] and high levels of pollutants in sardines, a small foraging fish, suggest that many species are suffering from varying levels of toxicity from environmental pollutants. The ecological consequences of the damage to aquatic ecosystems resulting from this observation raise concerns.

## 5. Conclusions

This study identified that all samples showed evidence of heavy metals. Although most elements were within limits established for safe consumption (Al, Ni, Cr, Cu, and Zn), barium, iron, and selenium exceeded the threshold set for children in at least one of the samples.

The second aim of this study was to investigate the effects of these elements’ amounts in the human health risk assessment of toddlers, children, adolescents, and adults. This investigation shows that the calculated *HQ* values indicated an adequate elemental content, with the exception of arsenic, which was the main component of *HI* values, and per se demonstrated inadequate metal content in all samples. Regardless of the *HQ* value for arsenic, cadmium, and lead, the mere presence of these contaminants is not acceptable in food products because there are no safe intake limits. Therefore, the heavy metal arsenic showed more serious carcinogenic risks; elements Cd and Cr also require attention.

The results found in this study indicate that it is not safe to consume canned sardines in Brazil, especially on a continued basis, regarding the toxic elemental content. These results shed light on this situation in Brazil and should motivate the authorities to enforce better surveillance of heavy metal contamination in canned products, such as sardines. Although this study focused on human health risk assessment, the findings may well also have a bearing on the country’s current legal framework.

## Figures and Tables

**Table 1:** Operating conditions for the microwave digestion system.

Step	Temperature (°C)	Pressure (Bar)	Ramp Time	Hold Time	Power (W)
1	100	30	1	5 min	1160
2	150	30	1	10 min	1160
3	50	25	1	1 min	0

**Table 2:** ICP-OES instrumental parameters.

Parameter	Setting
RF Power (W)	1250
Sample flow (L min^−1^)	0.35
Replicates	3
Plasma flow rate (L min^−1^)	12
Integration time (s)	5
Stabilization time (s)	20
Nebulization pressure(psi)	30
Plasma View	Axial
Analytes/λ	Al 167.079 nm, As 189.042 nm, Ba 455.403 nm, Cd 228.802 nm, Co 228.616 nm, Cr 283.563 nm, Cu 324.754 nm, Fe 259.940 nm, Ni 221.647 nm, Pb 220.353 nm, Se 196.090 nm, Zn 213.856 nm

**Table 3:** Elements, calibration equations (y = ax + b) *, limits of detection (LOD), limits of quantification (LOQ), and correlation coefficients (R^2^) obtained by external calibration.

Elements	Calibration Equations	LOD (mg/kg)	LOQ (mg/kg)	R^2^
Al	y = 135x − 0.8678	0.0044351	0.0147838	0.9989
As	y = 492.89x + 7.4355	0.0036706	0.0122353	0.9993
Ba	y = 812,405x + 7228.5	0.0001898	0.0006326	0.9994
Cd	y = 14,521x + 54.642	0.0006265	0.0020884	0.9996
Co	y = 6264.2x + 80.017	0.0009556	0.0031855	0.9993
Cr	y = 14,916x + 38.422	0.0008094	0.0026981	0.9997
Cu	y = 16,232x + 184.49	0.0017386	0.0057954	0.9995
Fe	y = 11,400x + 101.45	0.0169013	0.0563375	0.9994
Ni	y = 5542.7x + 66.307	0.0011056	0.0036853	0.9993
Pb	y = 1095.1x + 18.876	0.0050957	0.0169856	0.9994
Se	y = 376.77x + 5.7012	0.0052757	0.0175856	0.9994
Zn	y = 10,918x + 127.6	0.0031463	0.0104878	0.9994

* y = intensity; a = slope; x = concentration (mg/kg); b = intercept.

**Table 4:** Elemental content in canned sardines (mg/Tin 84 g).

Elements	Sardine in Oil (SO) mg/Tin 84 g					Sardine in Tomato Sauce (ST) mg/Tin 84 g					
	SO-G	SO-C	SO-O	SO-P	SO-Pa	ST-G	ST-C	ST-O	ST-P	ST-Pa	*p-*Value
Al	0.002 ^a,b^ ± 0.0002	0.0003 ^a^ ± 0.00002	0.002 ^a,b^ ± 0.0004	0.0008 ^a,b^ ± 0.0005	0.003 ^b^ ± 0.0002	0.002 ^a,b^ ± 0.0004	0.001 ^a,b^ ± 0.0002	0.008 ^c^ ± 0.0017	0.001 ^a,b^ ± 0.0001	0.002 ^a,b^ ± 0.0004	<0.0001
As	0.271 ^a,b^ ± 0.0091	0.256 ^a,b^ ± 0.0292	0.258 ^a,b^ ± 0.0189	0.377 ^b^ ± 0.0325	0.261 ^a,b^ ± 0.0271	0.318 ^a,b^ ± 0.0021 ^a^	0.323 ^a,b^ ± 0.0038 ^a^	0.224 ^a^ ± 0.0612	0.281 ^a,b^ ± 0.0142	0.207 ^a^ ± 0.0224	0.0146
Ba	0.037 ^a^ ± 0.0005	0.031 ^a^ ± 0.0145	0.033 ^a^ ± 0.0081	0.048 ^a^ ± 0.0004	0.020 ^a^ ± 0.0017	0.063 ^a^ ± 0.0202	0.061 ^a^ ± 0.0001	0.050 ^a^ ± 0.0291	0.066 ^a^ ± 0.0037	0.137 ^b^ ± 0.0007	<0.0001
Cd	0.00003 ^a^ ± 0.00001	0.00004 ^a^ ± 0.00002	0.00003 ^a^ ± 0.000001	0.00004 ^a^ ± 0.0000005	<LOD	0.00006 ^a^ ± 0.0001	<LOD	0.00002 ^a^ ± 0.0001	<LOD	0.002 ^b^ ± 0.00004	<0.0001
Cr	0.0015 ± 0.0002	0.001 ± 0.0018	0.002 ± 0.0013	0.002 ± 0.0009	0.001 ± 0.0007	0.0005 ± 0.0009	0.0009 ± 0.0015	0.001 ± 0.0010	<LOD	0.0003 ± 0.0040	0.9409
Cu	0.066 ^a^ ± 0.0099	0.054 ^a^ ± 0.0054	0.062 ^a^ ± 0.0001	0.220 ^b^ ± 0.0195	0.095 ^a,b^ ± 0.0152	0.092 ^a,b^ ± 0.0014	0.113 ^a,b^ ± 0.0261	0.062 ^a^ ± 0.0847	0.083 ^a,b^ ± 0.0182	0.099 ^a,b^ ± 0.0333	0.0261
Fe	2.320 ± 0.1423	1.466 ± 0.0833	1.996 ± 0.0487	2.820 ± 0.2194	1.945 ± 0.1648	2.611 ± 0.2045	2.568 ± 0.2962	3.507 ± 1.9340	1.463 ± 0.4138	1.827 ± 0.6371	0.1821
Ni	<LOD	0.009 ± 0.0170	<LOD	<LOD	<LOD	<LOD	<LOD	0.0002 ± 0.0084	<LOD	<LOD	>0.9999
Pb	0.0004 ^a^ ± 0.0001	0.0007 ^b^ ± 0.0006	0.0004 ^a^ ± 0.0002	0.0008 ^a^ ± 0.00003	0.00007 ^a^ ± 0.00001	0.0003 ^a^ ± 0.0002	0.0004 ^a^ ± 0.0002	0.0009 ^a^ ± 0.0012	0.0001 ^a^ ± 0.00002	0.0003 ^a^ ± 0.0003	<0.0001
Se	0.182 ± 0.0051	0.191 ± 0.0160	0.183 ± 0.0094	0.200 ± 0.0101	0.172 ± 0.0092	0.167 ± 0.0185	0.175 ± 0.0300	0.203 ± 0.0707	0.157 ± 0.0099	0.176 ± 0.0186	0.7933
Zn	0.036 ± 0.0055	0.027 ± 0.0060	0.029 ± 0.0053	0.039 ± 0.0016	0.017 ± 0.0006	0.033 ± 0.0026	0.038 ± 0.0095	0.033 ± 0.0119	0.020 ± 0.0029	0.023 ± 0.0055	0.0458

Note: Same letters in the same line mean no difference of the comparison analysis calculated by one-way ANOVA between samples elemental quantification. <LOD—analyte concentrations were below the limits of detection.

**Table 5:** Values of carcinogenic risk (*CR*) for exposure to As, Cd, Cr, Pb at ages 2, 5, 14, and 30 years in canned sardines SO, ST of the companies G, C, O, P, and Pa, considering an intake of 84 g/day.

Elements	Sample	Carcinogenic Risk (*CR*)			
		Toddlers (Aged 1–3 Years)	Children (Aged 3–10 Years)	Adolescents (Aged 10–18 Years)	Adults (Aged > 18–65 Years)
Arsenic	SO-G	1.45 × 10^−2^	7.52 × 10^−3^	3.34 × 10^−3^	2.48 × 10^−3^
	SO-C	1.37 × 10^−2^	7.10 × 10^−3^	3.16 × 10^−3^	2.34 × 10^−3^
	SO-O	1.38 × 10^−2^	7.16 × 10^−3^	3.18 × 10^−3^	2.36 × 10^−3^
	SO-P	2.01 × 10^−2^	1.05 × 10^−2^	4.65 × 10^−3^	3.45 × 10^−3^
	SO-Pa	1.39 × 10^−2^	7.24 × 10^−3^	3.22 × 10^−3^	2.39 × 10^−3^
	ST-G	1.70 × 10^−2^	8.83 × 10^−3^	3.92 × 10^−3^	2.91 × 10^−3^
	ST-C	1.73 × 10^−2^	8.96 × 10^−3^	3.98 × 10^−3^	2.96 × 10^−3^
	ST-O	1.20 × 10^−2^	6.22 × 10^−3^	2.76 × 10^−3^	2.05 × 10^−3^
	ST-P	1.50 × 10^−2^	7.80 × 10^−3^	3.46 × 10^−3^	2.57 × 10^−3^
	ST-Pa	1.11 × 10^−2^	5.74 × 10^−3^	2.55 × 10^−3^	1.90 × 10^−3^
Cadmium	SO-G	6.52 × 10^−6^	3.39 × 10^−6^	1.50 × 10^−6^	1.12 × 10^−6^
	SO-C	8.69 × 10^−6^	4.51 × 10^−6^	2.01 × 10^−6^	1.49 × 10^−6^
	SO-O	6.52 × 10^−6^	3.39 × 10^−6^	1.50 × 10^−6^	1.12 × 10^−6^
	SO-P	8.69 × 10^−6^	4.51 × 10^−6^	2.01 × 10^−6^	1.49 × 10^−6^
	SO-Pa	-	-	-	-
	ST-G	1.3 × 10^−5^	6.77 × 10^−6^	3.01 × 10^−6^	2.23 × 10^−6^
	ST-C	-	-	-	-
	ST-O	4.35 × 10^−6^	2.26 × 10^−6^	1.00 × 10^−6^	7.45 × 10^−7^
	ST-P	-	-	-	-
	ST-Pa	4.35 × 10^−4^	2.26 × 10^−4^	1.00 × 10^−4^	7.45 × 10^−5^
Chromium	SO-G	2.67 × 10^−5^	1.40 × 10^−5^	6.16 × 10^−6^	4.58 × 10^−6^
	SO-C	1.83 × 10^−5^	9.50 × 10^−6^	4.23 × 10^−6^	3.14 × 10^−6^
	SO-O	4.27 × 10^−5^	2.20 × 10^−5^	9.86 × 10^−6^	7.33 × 10^−6^
	SO-P	3.56 × 10^−5^	1.90 × 10^−5^	8.22 × 10^−6^	6.11 × 10^−6^
	SO-Pa	1.78 × 10^−5^	9.30 × 10^−6^	4.11 × 10^−6^	3.05 × 10^−6^
	ST-G	8.9 × 10^−6^	4.60 × 10^−6^	2.05 × 10^−6^	1.53 × 10^−6^
	ST-C	1.6 × 10^−5^	8.30 × 10^−6^	3.70 × 10^−6^	2.75 × 10^−6^
	ST-O	2.15 × 10^−5^	1.10 × 10^−5^	4.97 × 10^−6^	3.69 × 10^−6^
	ST-P	-	-	-	-
	ST-Pa	5.93 × 10^−6^	3.10 × 10^−6^	1.37 × 10^−6^	1.02 × 10^−6^
Lead	SO-G	1.21 × 10^−7^	6.30 × 10^−8^	2.79 × 10^−8^	2.08 × 10^−8^
	SO-C	2.12 × 10^−7^	1.10 × 10^−7^	4.89 × 10^−8^	3.63 × 10^−8^
	SO-O	1.21 × 10^−7^	6.30 × 10^−8^	2.79 × 10^−8^	2.08 × 10^−8^
	SO-P	2.42 × 10^−7^	1.30 × 10^−7^	5.59 × 10^−8^	4.15 × 10^−8^
	SO-Pa	2.12 × 10^−8^	1.10 × 10^−8^	4.89 × 10^−9^	3.63 × 10^−9^
	ST-G	9.08 × 10^−8^	4.70 × 10^−8^	2.10 × 10^−8^	1.56 × 10^−8^
	ST-C	1.21 × 10^−7^	6.30 × 10^−8^	2.79 × 10^−8^	2.08 × 10^−8^
	ST-O	2.72 × 10^−7^	1.40 × 10^−7^	6.29 × 10^−8^	4.67 × 10^−8^
	ST-P	3.03 × 10^−8^	1.60 × 10^−8^	6.99 × 10^−9^	5.19 × 10^−9^
	ST-Pa	9.08 × 10^−8^	4.70 × 10^−8^	2.10 × 10^−8^	1.56 × 10^−8^

## Data Availability

Data will be available upon reasonable request to the corresponding author.
